# Typhoid ulcer causing life-threatening bleeding from Dieulafoy's lesion of the ileum in a seven-year-old child: a case report

**DOI:** 10.1186/1752-1947-4-171

**Published:** 2010-06-03

**Authors:** Rajan Fuad Ezzat, Hiwa A Hussein, Trifa Shawkat Baban, Abbas Tahir Rashid, Khaled Musttafa Abdullah

**Affiliations:** 1 Department of Surgery, Sulaimanyah Teaching Hospital, Sulaimanyah, Iraq; 2 Department of Medicine, Sulaimanyah Teaching Hospital, Sulaimanyah, Iraq; 3 Department of Pathology, Sulaimanyah Teaching Hospital, Sulaimanyhah, Iraq

## Abstract

**Introduction:**

We describe a case of rare complication of typhoid fever in a seven-year-old child and review the literature with regard to other rare causes of bleeding per rectum. Dieulafoy's lesion is an uncommon but important cause of recurrent gastrointestinal bleeding. Dieulafoy's lesion located extragastrically is rare. We report a case of typhoid ulcer with Dieulafoy's lesion of the ileum causing severe life-threatening bleeding and discuss the management of this extremely uncommon entity.

**Case presentation:**

As a complication of typhoid fever, a seven-year-old Kurdish girl from Northern Iraq developed massive fresh bleeding per rectum. During colonoscopy and laparotomy, she was discovered to have multiple bleeding ulcers within the Dieulafoy's lesion in the terminal ileum and ileocecal region.

**Conclusion:**

Although there is no practical way of predicting the occurrence of such rare complications, we emphasize in this case report the wide array of pathologies that can result from typhoid fever.

## Introduction

Typhoid fever and paratyphoid fever is a systemic infection caused by *Salmonella enterica*, including *S. enterica *serotype Typhi (S. typhi) and serotype Paratyphi (S. paratyphi). Enteric fever is a faecal-oral transmissible disease and thus occurs in an environment with overcrowding, poor sanitation and untreated water [1].

Complications occur in 10 to 15% of patients and are particularly likely in patients who have been ill for more than two weeks. Many complications have been described, of which gastrointestinal bleeding, intestinal perforation, and typhoid encephalopathy are the most important [1]. Gastrointestinal bleeding is the most common symptom and it occurs in up to 10% of patients. It results from the erosion of a necrotic Peyer's patch through the wall of an enteric vessel. In the majority of cases, the bleeding is slight and resolves without the need for blood transfusion. In 2% of cases, however, bleeding is clinically significant and can be rapidly fatal if a large vessel is involved. Intestinal (usually ileal) [1,2] perforation is the most serious complication of the disease and it occurs in 1 to 3% of hospitalized patients [1-3].

Intestinal bleeding in typhoid fever usually occurs from the ulcers in the ileum or the proximal colon, and the most common colonoscopic manifestations are multiple variable-sized punched-out ulcerations. The shape of the ulcers is usually ovoid with the longest diameter parallel to the long axis of the gut, so that stricture formation does not occur after healing. The edges are soft, swollen and irregular, but not undermined. The floor is usually smooth and is formed by the muscular coat. Near the ileocecal valve, where perforation occurs more commonly, ulcers become deeper than elsewhere [2]. Although uncommon, sporadic cases of typhoid fever still occur.

Involvement of the small intestine is nearly universal [1]. Hemorrhage and intestinal perforation are the two major complications of small intestinal typhoid infection. Therapy for hemorrhaged small intestine in typhoid fever is initially supportive, consisting of blood transfusions and administration of antibiotics. In massive or recurrent hemorrhage, consideration is given to surgical resection of the involved small-intestinal segment. Operative management of the complications of small intestinal typhoid infection has a high associated mortality rate [1,2]. Here we report a case of typhoid ileitis with massive hemorrhage from diffuse punched-out ulcerations and erosions in the terminal ileum successfully treated by surgical excision of the diseased part.

### Case presentation

A seven-year-old Kurdish girl from northern Iraq presented to our hospital with fever, abdominal pain, nausea, vomiting and diarrhea for one week duration, followed by fresh bleeding per rectum after 10 days from her illness for three days before her admission. She had history of neither chronic medical disease nor surgical operation. Her physical examination on admission revealed the following: pallor, BP = 80/50 mmhg, PR = 97 b/m, rapid respiration (shock state), temperature = 40.2 ˚C. Her abdominal examination revealed mild splenomegaly with diffuse abdominal tenderness mainly in the right lower quadrant. Blood profile showed a hemoglobulin of 7.1 g/dl, white blood cell of 4500/ml, and agglutinations for *O. salmonella *antigens at 1:160. Culture from our patient grew *Salmonella typhi*. An abdominal ultrasound of our patient revealed splenomegaly.

Once she was admitted to our hospital's emergency department, supportive measures were performed on her, including intravenous line, blood transfusion (five pints) and the administration of broad spectrum antibiotics in the form of third generation cephalosporin (Ceftriaxone) 1 gm daily. An urgent colonoscopy was arranged for our patient, which showed that her colon was full of fresh blood. After suction and irrigation the terminal ileum of our patient was intubated, which revealed multiple variable size punched-out ileal ulcers up to 25 cm from the ileocecal valve, as well as ulceration of the ileal mucosa with characteristic dilated aberrant submucosal vessel that erodes the overlying epithelium.

**Figure 1:**
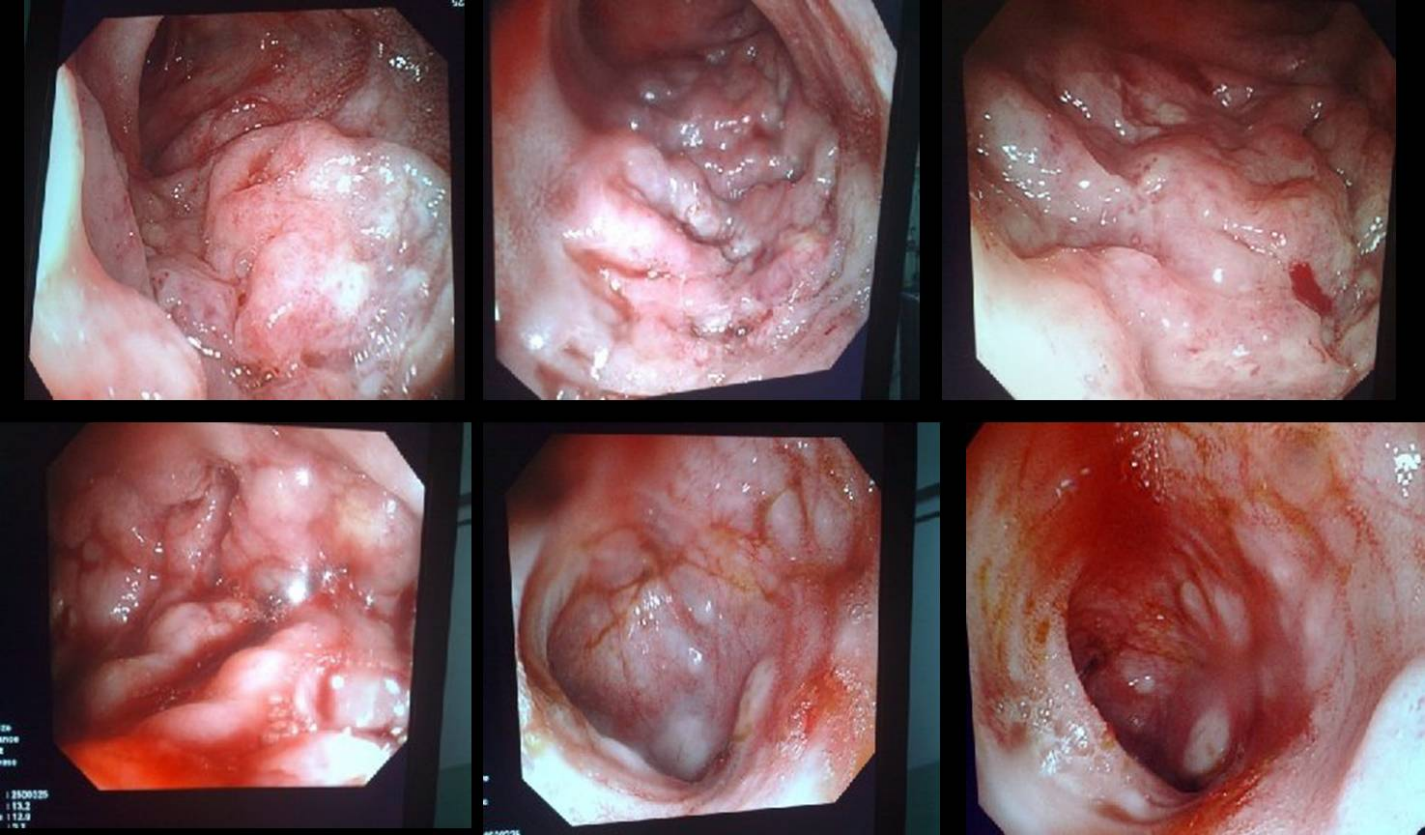
**Diffuse terminal ileal ulceration and angiomal formtion with oozing hemorrhage from these ulcers**.

**Figure 2:**
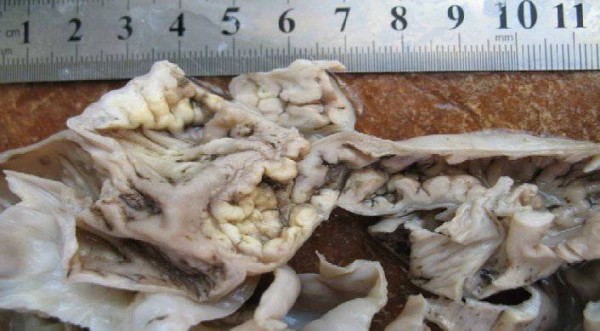
**The surgical specimen after resection**.

**Figure 3:**
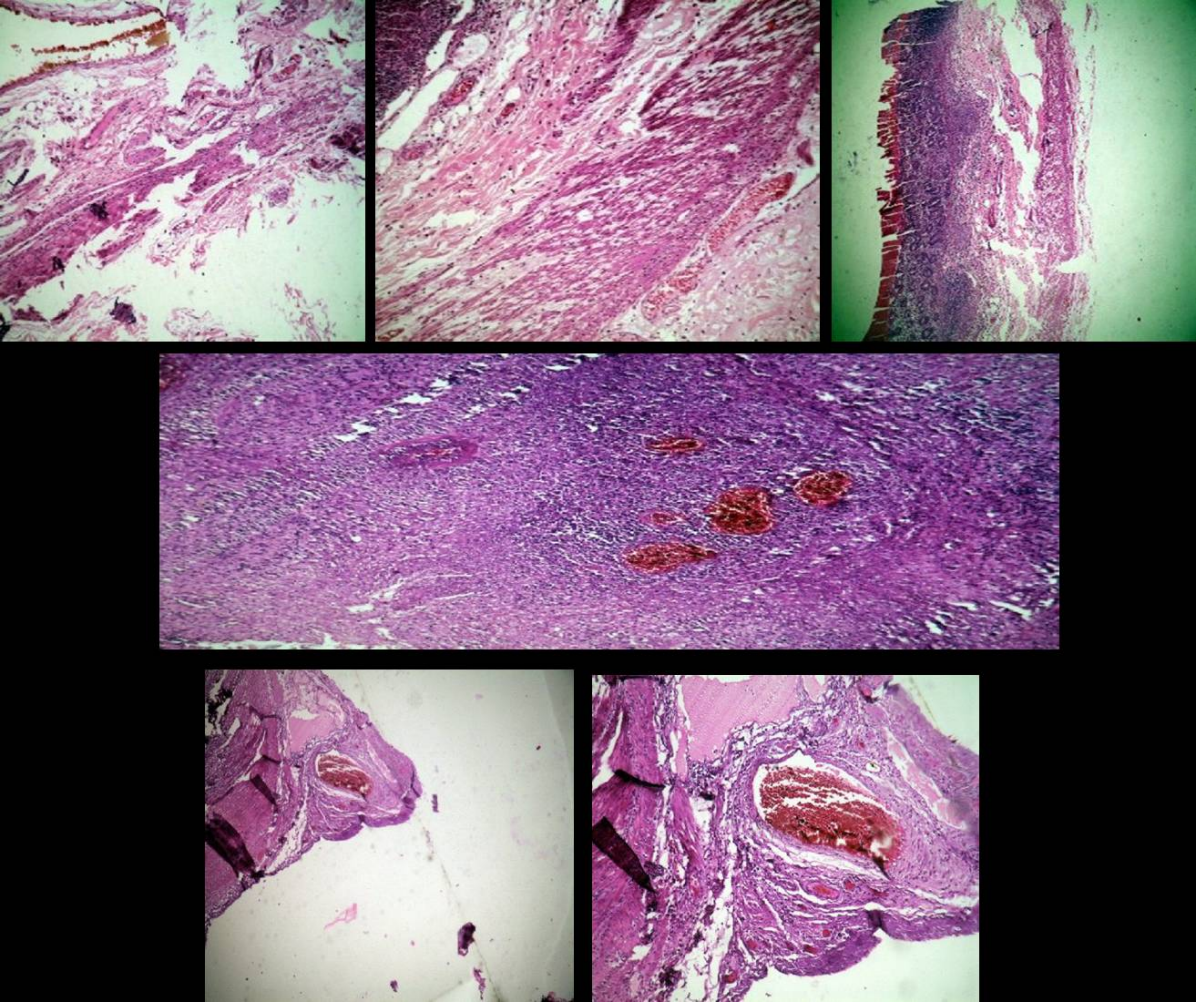
**The arteriovenous malformation in different sections and views**.

In the absence of a primary ulcer, Dieulafoy's lesion was seen in the terminal ileum with oozing hemorrhage from this lesion 10 cm away from the ileocecal valve (Figure 1). The bleeding could not be controlled by endoscopic hemostasis using thermal coagulation or any other endoscopic intervention. A decision was made accordingly for urgent explorative laparotomy to save her life. An ileocolectomy (emergency limited segmental resection for a known bleeding source) was also done on our patient (Figure 2). Histology revealed this to be of the Dieulafoy type of lesion in the distal ileum (Figure 3).

Our patient had a very smooth post-operative course. Her hematochezia disappeared the next day and she was discharged in good health within eight days. One month later, she was completely asymptomatic. The biopsy specimen of the distal 25 cm of her ileum located 20 cm from her right colon had numerous irregular punched out-ulcers, extensive inflammation, and focal suppuration infiltrating mucosa and submucosa.

Macroscopic examination revealed a vascular malformation with a visible clot within. Microscopy revealed a lesion comprising of thick-walled arteries and veins representing an arteriovenous malformation (AVM). A degree of thrombosis and recanalization was also observed. The appearances were those of an AVM of the Dieulafoy type. Ulcers were also revealed, and some of them were deep. Mixed inflammatory cell infiltrate predominated the ulcers without caseous necrosis. The mesenteric lymph nodes of our patient revealed reactive sinus hyperplasia (Figure 3).

## Discussion

The percentage of patients that presented with lower gastrointestinal bleeding (GIB) in patients with typhoid fever, whether clinically suspected or blood culture positive, was 2%. This is much lower than that reported in the literature (10%) [1]. Most patients with lower GIB were young [2]. Endoscopic demonstration of colonic and terminal ileum lesions of typhoid by colonoscopy is scarcely reported in the medical literature, as these examinations are only advised when the etiological diagnosis is not yet established.

Ulcerations generally occur in the terminal ileum, cecum and the ascending colon, and rarely in the left side of the colon [4]. Dieulafoy lesions rarely cause gastrointestinal hemorrhage. These lesions were first identified by Gallard in 1884 and formally described by Dieulafoy in 1897. Macroscopically, AVM comprises a small pea-sized lesion appearing as a mucosal defect with an artery protruding from its base.

Histologically, a thick-walled arterial vessel is seen. This is larger than the surrounding submucosal vessels and runs below the muscularis mucosae. A similar appearance has been reported throughout the gastrointestinal tract. Although the pathogenesis is unclear, the lesion is believed to be congenital in origin. Endoscopic diagnosis of extragastric Dieulafoy's lesion can be difficult because of its small size and obscure location. Increased awareness and careful and early endoscopic evaluation following the bleeding episode are the key to accurate diagnosis [5]. In a large series from a tertiary care center in India, out of 900 cases of upper GIB, only six (0.67%) were caused by DL. The lesion was located within 6 cm of the gastroesophageal junction in all cases [1].

Extragastric DLs are uncommon. In a review of over 100 cases of DLs, Veldhuyzen found no lesion of the duodenum [6]. Similar lesions have also been described in the esophagus [7-9], duodenum, jejunum, colon and rectum [8,10-12]. Extragastric DLs have been identified more frequently in recent years because of increased awareness of the condition [7,8]. In a large series of 89 patients with DLs, the lesions were extragastric in a third of the cases.

The duodenum was the most common location (18%) of extragastric DLs, followed by the colon (10%), jejunum (2%) and the esophagus (2%) [8]. The pathology of the lesion is essentially the same throughout the gastrointestinal tract and is caused by an abnormally large calibre persistent tortuous submucosal artery [13].

## Conclusion

The endoscopic criteria proposed to define DL are: 1) active arterial spurting or micropulsatile streaming from a minute mucosal defect or through normal surrounding mucosa; 2) visualization of a protruding vessel with or without active bleeding within a minute mucosal defect or through normal surrounding mucosa; and 3) fresh, densely adherent clot with a narrow point of attachment to a minute mucosal defect or to normal appearing mucosa [14].

Meanwhile, several surgical options are used in the management of patients with lower intestinal bleeding: 1) emergency limited segmental resection for a known bleeding source in continued severe bleeding (directed segmental resection); 2) elective segmental resection for a known bleeding source such as adenocarcinoma of the colon or for rebleeding from a known lesion such as a colon diverticulum; 3) emergency segmental colon resection for an unknown bleeding source (blind segmental resection); and 4) emergency total abdominal colectomy with ileorectal anastomosis (subtotal colectomy) for an unknown bleeding location.

Several criteria have been used to recommend surgery for patients with acute lower intestinal bleeding. Transfusion requirements of >4 units during 24 hours and before 10 units overall have been used as indicators for surgical intervention. These data support the importance of pursuing an aggressive approach to pre-operative localization of the bleeding source.

### Consent

Written informed consent was obtained from the parents/guardian of our patient for publication of this case report and any accompanying images. A copy of the written consent is available for review by the Editor-in-Chief of this journal.

## Competing interests

The authors declare that they have no competing interests.

## Authors' contributions

RFE collected, analyzed and interpreted our patient's data, and assisted in the endoscopy and performed the surgery of our patient. HH performed the endoscopy and assisted in interpreting our patient's data. TSB performed the histological examination of the surgical specimen and assisted in interpreting our patient's data. ATR assisted in the operation and in analyzing our patient's data. KMA received our patient and assisted in collecting our patient's data. All authors read and approved the final manuscript.
